# Research on the relationship between population distribution pattern and urban industrial facility agglomeration in China

**DOI:** 10.1038/s41598-023-43376-4

**Published:** 2023-09-27

**Authors:** Peng Zeng, Cheng Zong

**Affiliations:** 1 School of Ethnology and Sociology, Guangxi Minzu University, Nanning, 530006 Guangxi China; 2 School of Economics, Guangxi Minzu University, Nanning, 530006 Guangxi China

**Keywords:** Population dynamics, Scientific data, Socioeconomic scenarios, Sustainability

## Abstract

Investigating the impact of industrial facility agglomeration on population distribution provides valuable insights for advancing urban and regional development, as well as aiding in planning, forecasting, and achieving regional equilibrium. However, there remains a notable gap in understanding the influence and mechanisms of industrial facility agglomeration on population distribution, particularly when considering different industry types and diverse regions comprehensively. Additionally, conventional panel data used to assess industrial facility agglomeration are constrained by limitations in coverage and timeliness. In contrast, Point of Interest (POI) data offers a superior solution with its real-time, fine-grained, and innovative advantages. This study utilizes real-time and fine-grained POI data in conjunction with the LandScan population raster dataset to precisely assess industrial facility agglomeration in 352 administrative units at the prefecture level and above in China. The key findings of this research can be summarized as follows: (1) factors influencing urban population growth rates have evolved, with increased significance attributed to Government Agencies and Social Groups, alongside a consistent impact from Science, Education, and Cultural Services. (2) The correlation between industrial facility agglomerations and population growth rates displayed linear relationships in 2015 and 2021, with varying strengths and directional shifts. (3) Regional disparities in industrial facility agglomeration patterns underscore the necessity for customized strategies to optimize industrial structures, foster innovation-driven sectors, and promote sustainable population growth.

## Introduction

In recent decades, rapid economic development and urbanization have emerged as global phenomena with far-reaching implications for population distribution and industrial dynamics^1^. These transformative processes extend beyond the borders of any single nation and have profound consequences for societies and economies worldwide^2^. This study delves into the intricate interplay between these forces, focusing on China, a nation that has experienced remarkable demographic and economic shifts between 2015 and 2021. China's rapid economic development and urbanization over the past few decades have profoundly affected population distribution and industrial development. Between 2015 and 2021, China experienced significant demographic and economic shifts characterized by a consistent yet declining population growth rate^3^. Concurrently, the nation experienced dynamic shifts in its economic landscape, including a transition towards a more service-oriented economy, intensified technological innovation, and evolving trade dynamics. These economic developments posed significant challenges and opportunities, influencing labor markets, income distribution, and industrial structure^4^. Recognizing the multifaceted nature of these changes, the Chinese government embarked on policy revisions to address demographic and economic concerns. This comprehensive approach centralizes population dynamics and urban industrial development within the broader framework of social and economic development^5^.

Furthermore, China's population distribution displayed notable regional disparities, rapid urbanization, an aging demographic, and decelerated growth during this period. Eastern coastal regions witnessed high population densities due to an influx of rural laborers and migrants, while western regions experienced lower population densities^6^. Urbanization resulted in declining rural populations and an expanding urban–rural divide^7,8^. The aging population posed challenges to social security and the labor market, while internal population mobility transformed due to new policies, reforms, and rural economic development^9^. Notably, despite introducing the two-child policy in 2015, persistently low fertility rates persisted, influenced by cultural beliefs and economic conditions^10^.

Given the dynamic interplay between demographic shifts and industrial development, this study focuses on the intricate relationship between urban industrial facility agglomeration and population distribution in China from 2015 to 2021. The primary objectives are to (1) unveil distinctive population distribution patterns and industrial facility agglomeration features across various regions, (2) investigate the diverse impacts of different industrial facility agglomerations on urban population size, and (3) scrutinize the intricate relationship between industrial facility agglomeration and population distribution to develop a more comprehensive theoretical framework.

This study employs various quantitative analysis techniques to rigorously investigate the relationship between population distribution patterns and urban industrial facility agglomeration in China from 2015 to 2021. Kernel density analysis is initially employed to describe the spatial distribution characteristics of the population and industrial facility agglomeration^11^. Subsequently, spatial autocorrelation analysis is utilized to assess the degree of spatial association between population distribution and industrial facility agglomeration. Finally, multiple regression analysis is applied to unveil the relationship between industrial facility agglomeration and urban population size^12^ and to evaluate the heterogeneous impact of different industrial facility agglomerations on urban population size^13^.

## Literature review

Industrial facility agglomeration refers to the spatial concentration of industrial activities^14^, with its intellectual roots tracing back to the seminal works of Marshall^15^ and Weber^16^. However, it gained significant prominence and theoretical grounding within the framework of New Economic Geography, mainly due to the influential contributions of Krugman^17^, particularly his introduction of the concept of increasing returns to scale, which has fundamentally shaped our understanding of urban industrial facility agglomeration.

The significance of urban industrial facility agglomeration extends across multiple dimensions of urban development. Empirical evidence from Nielsen et al.^18^ underscores its role in enhancing production efficiency. Furthermore, studies by Liao and Li^19^ emphasize its contribution to fostering innovation, bolstering urban competitiveness, and catalyzing regional development. Indeed, urban industrial facility agglomeration plays a pivotal role in comprehending the dynamics of urban economic growth, optimizing spatial structures, fortifying competitive advantages, and advancing the cause of sustainable development, as highlighted in research conducted by Huang et al.^20^ and Chen et al.^21^.

The scholarly exploration of industrial facility agglomeration and its relationship with urban population size has encompassed diverse perspectives and research dimensions. Previous investigations have unraveled the intricate interplay between population mobility and urban industrial development, examined the influence of population distribution on urban industrial structure, and assessed the role of urban industrial facility agglomeration in shaping population distribution patterns^22^.

Primarily, research in this domain has delved into the interaction between industrial facility agglomeration and urban population size, frequently employing classical models such as the push–pull population migration theory^23^. A notable finding from this body of research highlights the pivotal role of human capital as a driver of industrial advancement. Population agglomeration, initially driven by economic development, encounters constraints owing to increasing demands for human capital^24^. Secondly, investigations have scrutinized the influence of urban population size on industrial facility agglomeration. Within this context, labor dynamics have exhibited a "cumulative causation" effect, with the net impact of urban scale on industrial facility agglomeration being significantly positive in the short term but potentially diminishing over the medium to long term^25^. Finally, research efforts have explored the mechanisms underpinning urban population agglomeration. While industrial facility agglomeration drives population agglomeration, uncertainties linger regarding its impact on urban population size^13,26^. For instance, the agglomeration of secondary industry facilities has boosted population size growth, while tertiary industries and industrial structure sophistication may suppress population growth^27^. Additionally, industrial facility agglomeration can sometimes lead to agglomeration diseconomies, potentially hindering productivity growth^28,29^.

However, it is essential to acknowledge that existing research in this field exhibits certain limitations, primarily related to geographical scope and research periods. Most studies have focused on national-level or representative city-level analyses, often overlooking regional disparities^30,31^. Furthermore, limited attention has been paid to the crucial period between 2015 and 2021, during which China witnessed significant shifts in population distribution patterns and industrial facility agglomeration characteristics^32^.

This study contributes significantly to the field by introducing innovative perspectives and addressing noteworthy research gaps. Specifically, it departs from conventional panel data techniques and employs groundbreaking methods utilizing POI data to measure industrial facility agglomeration. This approach provides real-time and granular data, enabling precise evaluations of industrial concentration within diverse urban settings. By effectively overcoming the limitations of traditional data, such as inadequate coverage, delayed updates, and the absence of fine-grained information, this pioneering application of POI data offers fresh insights and invaluable analytical tools for comprehending urban development and facilitating effective urban planning. Furthermore, this study delves into the intricate relationship between various forms of urban industrial facility agglomeration and urban population growth rates across 352 administrative units at or above the prefecture level throughout the country, focusing on the eastern, central, and western regions. This unique and meticulous regional approach delivers a comprehensive understanding and comparative analysis of how urban industrial facility agglomeration influences population growth across distinct regions, uncovering the nuances and distinctive attributes of the eastern, central, and western regions^33^. Consequently, this study equips local governments and urban planners with targeted development strategies and policy recommendations tailored to their contexts^34^.

## Methods and data

**Figure 1:**
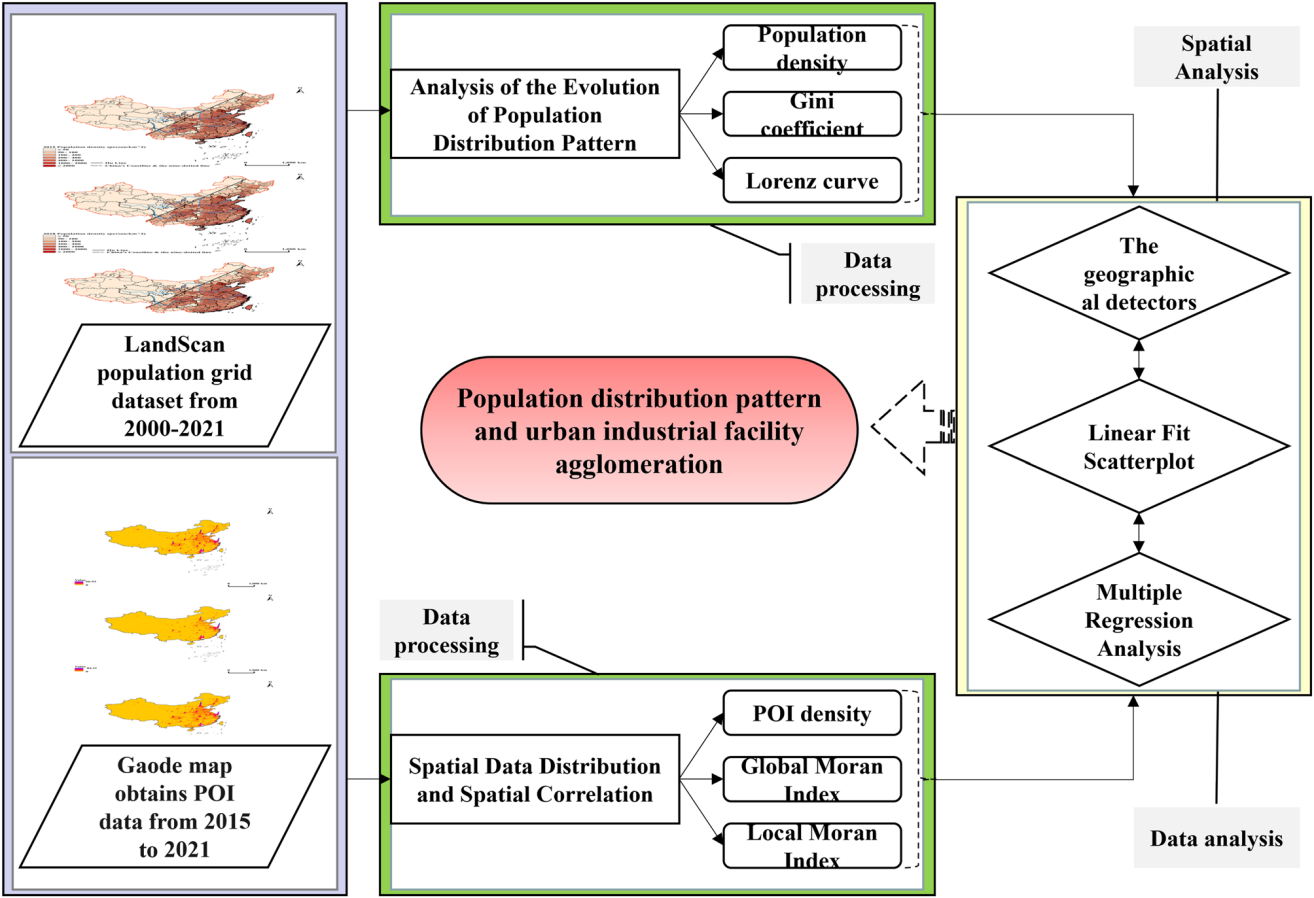
Research method and data processing flowchart. A flowchart illustrating the research methodology and data processing steps. Created using Microsoft® PowerPoint® 2019MSO (Version 2308 Build 16.0.16731.20182) 64-bit.

This study employs statistical and spatial analysis techniques to explore the evolution of population distribution patterns and their relationship with industrial facility agglomeration. The overall method consists of four main steps: data collection, data processing, analysis, and interpretation of results. The methodological innovation of this research lies in integrating multiple techniques, such as the Gini coefficient, Moran's I, the geographical detectors, and multiple regression analysis, which together provide a comprehensive understanding of the spatial distribution and correlation of data. This approach is valuable in guiding decision-making in geography, urban planning, and environmental protection. The overall technical flow chart of this paper is shown in Fig. 1.

### Analysis of the evolution of population distribution pattern

The Gini coefficient is a statistical measure employed to evaluate inequality levels and can be applied to quantify the disparities in population distribution patterns. In this study, we rank the population density of each province and the autonomous region at the prefecture-level and higher administrative units and establish a Lorenz curve for population distribution using cumulative percentages of population and area. Specifically, we organize all spatial units within region $Z$ in descending order based on their population density, with the sequence of spatial units represented as $\left( {i = 1,2,3 \ldots n} \right)$. Subsequently, we calculate the proportion of the population in unit $i$ relative to the total population ($P_{i}$) and the proportion of the area within the unit relative to the total area ($W_{i}$). The Gini coefficient ($G_{Z}$) is determined using the following formula:

1 $$ G_{Z} = 1 + \mathop \sum \limits_{i = 1}^{n} P_{i} W_{i} - 2\mathop \sum \limits_{i = 1}^{n} P_{i} Q_{i} $$

In this equation, $Q_{i} = \sum\nolimits_{k = 1}^{i} {W_{k} }$ denotes the cumulative percentage of the area.

### Spatial data distribution and spatial correlation

Moran's I and the geographical detectors are tools utilized to study the spatial distribution and correlation of spatial data. Moran's I is a spatial autocorrelation statistical method that measures spatial data's degree of spatial clustering^35^. It reflects whether the observed values in a region have spatial correlation, i.e., whether they are close or far apart in space. The larger the value of Moran's I, the stronger the spatial correlation, and vice versa. The geographical detectors can further explore the spatial relationships behind Moran's I and determine these relationships' causes and influencing factors^36^. It helps researchers identify hotspot areas in space, i.e., areas with significant spatial clustering properties, while also revealing spatial heterogeneity, i.e., the differences and diversity between different regions in space. The core idea behind the geographical detectors is based on the assumption that if an independent variable significantly impacts a dependent variable, then the spatial distribution of the independent and dependent variables should be similar. Therefore, Moran's I and the geographical detectors can complement each other and be used together to gain a deeper understanding of the distribution and correlation of spatial data, providing support and guidance for decision-making in fields such as geography, urban planning, and environmental protection.

Spatial autocorrelation refers to the degree of similarity between adjacent regions in geographic space in terms of a particular attribute. The primary purpose of spatial autocorrelation is to reveal the mutual relationships between spatial units and the patterns of spatial structure^37^. The calculation methods for spatial autocorrelation include global and local spatial autocorrelation. Based on the results of the analysis of the impact of industrial facility agglomeration on population distribution patterns, this article used global Moran's I to reveal the overall spatial correlation trend in China^38^ and local Moran's I to clarify the spatial agglomeration degree and heterogeneity of each prefecture-level administrative unit^39^.

The z-score standardization method is commonly used in spatial autocorrelation analysis because it can better handle customarily distributed data. Standardizing data using the z-score method converts data into dimensionless data, and the formula for z-score standardization is as follows:

2 $$ z = \left( {x - \mu } \right) / \sigma $$

In this formula, $z$ represents the standardized data, $x$ represents the original data, $\mu$ represents the mean of the original data, and $\sigma$ represents the standard deviation of the original data^40^.

The geographical detectors are based on the theory of spatial heterogeneity and diagnose a research object's spatial or attribute heterogeneity or coupling to reveal the mechanisms and major driving factors^41^. Using factor detectors, this study analyzed the explanatory power of various industrial facility agglomerations for the population growth rate of prefecture-level and higher administrative units, and the analytical model is as follows:

3 $$ q = 1 - \frac{1}{{K\mu^{2} }}\mathop \sum \limits_{h = 1}^{R} N_{h} \mu_{h}^{2} $$

In this formula, $N_{h}$ and $\mu_{h}^{2}$ represent the sample size and variance of layer $h\left( {h = 1, 2, \ldots , R} \right)$, $K$ represents the total sample size, and $q$ represents the explanatory power of each detector factor for the population growth rate of prefecture-level and higher administrative units, ranging from 0 to 1. A larger $q$ value indicates a stronger explanatory power of the detector factor for its spatial distribution, while a smaller $q$ value indicates weaker explanatory power^42^. In this study, 12 detector factors representing industrial facility agglomeration were selected: Catering Services (A), Famous tourist sites (B), Company (C), Shopping service (D), Financial insurance service (E), Science, education and cultural services (F), Business residence (G), Domestic services (H), Sports and Leisure Services (I), Healthcare services (J), Government agencies and social groups (K), and Accommodation service (L). The k-means classification algorithm was used to classify these factors^22,43^.

### Explanatory models and variables

This study employed multiple regression analysis to investigate the relationship between urban population growth rate and agglomeration of industrial facilities. Multiple regression analysis is a standard statistical technique used to examine the effects of multiple independent variables on a dependent variable while controlling for other variables. The dependent variable in this study was the urban population growth rate, and the independent variables were the agglomeration levels of 12 types of urban industrial facilities^44^. A residual normality test, variance inflation factor test, and stepwise regression analysis were conducted to ensure the validity and accuracy of the model. The hypothesis that residuals follow a normal distribution was initially confirmed by observing *p* values and figures. Subsequently, multicollinearity issues were examined by computing each independent variable's variance inflation factor. Lastly, stepwise regression analysis was conducted to obtain a final regression model that contains significant independent variables. This approach helped to identify the most influential independent variables, eliminate insignificant ones, and improve the model's predictability and interpretability. The benchmark econometric model used in this study is as follows:

4 $$ growth_{it} = \beta_{0} A_{{IA_{it} }} + \beta_{1} B_{{IA_{it} }} + \ldots + \beta_{11} L_{{IA_{it} }} + \varepsilon_{it} $$

Here, $growth_{it}$ represents the population growth rate in region $i$ during period $t$, and $A_{{IA_{it} }}$ represents the agglomeration level of industry type $A$ in region $i$ and period $t$. A total of 12 industry types $\left( {A, B, \ldots , L} \right)$ were examined as independent variables^45^.

### Data source and calculation method

The population data utilized in this study were obtained from the LandScan population grid dataset with a resolution of 1 km from 2015 to 2021. The LandScan population dataset was developed by the Oak Ridge National Laboratory and provided by East View Cartographic. LandScan is a social standard for publishing global population data using innovative GIS and remote sensing methods. It is the most accurate and reliable global population dynamic statistical analysis database based on geographical location, with distribution models and optimal resolution.

POI, an abbreviation for "Point of Interest," refers to any meaningful point on a map that is not geographically significant, such as shops, bars, gas stations, hospitals, and stations^46^. The POI dataset used in this study from 2015 to 2021 was obtained by crawling the Amap API interface. In the specific operation, it was necessary to first apply for an Amap API KEY and obtain the "POI classification code table." The final results of this study were generated by separate tables for different POI types, with table fields including longitude, latitude, number of retrievals, POI category name, POI sub-category name, POI name, POI code, and administrative unit name, totaling eight items. The specific POI data information is shown in Table 1.

**Table 1:** Number of POIs in different types of industries from 2015 to 2021.

Industrial type	Code	2015 quantity	2018 quantity	2021 quantity	Changes in data
Catering services	A	1,522,185	5,334,137	5,219,012	
Famous tourist sites	B	94,674	213,586	212,220	
Company	C	1,519,957	1,284,900	3,183,189	
Shopping service	D	3,279,475	2,926,294	12,108,270	
Financial insurance service	E	470,121	781,477	769,391	
Science, education and cultural services	F	641,541	1,486,632	1,284,764	
Business residence	G	6,48,961	1,007,888	999,398	
Domestic services	H	1,578,650	4,453,539	4,367,347	
Sports and leisure services	I	327,616	757,104	743,638	
Healthcare services	J	728,127	1,430,188	1,399,093	
Government agencies and social groups	K	1,018,948	1,503,121	1,491,360	
Accommodation service	L	353,076	803,661	792,569	
Total	All	12,183,331	21,982,527	32,570,251	

Our study necessitated the seamless integration of two diverse datasets: POI data and population raster data. This integration was executed with precision using ArcGIS software, encompassing a structured, step-by-step approach within a geospatial context.

*Step 1* Our journey commenced with population data initially in raster format. Employing ArcGIS, we conducted georeferencing for spatial alignment, data extraction for relevant information, and, when required, conversion to vector format. This transformation yielded vector polygons representing precise geographic regions. *Step 2* Concurrently, the POI dataset underwent rigorous preprocessing using ArcGIS. Data cleansing rectified inconsistencies, spatial clustering optimized data representation, and attribute refinement enhanced overall data accuracy. *Step 3* ArcGIS facilitated spatial joins, linking the population data (in vector polygons) with the POI dataset. This step precisely associated each POI with its corresponding geographic area. *Step 4* The final phase utilized ArcGIS's toolkit to merge and attribute relevant data fields from both datasets systematically. This meticulous process ensured the accurate linkage of each POI to the geographic area defined by the population data.

## Research result

### Evolution trend of population distribution pattern in China

The changing trend of China's population distribution pattern is closely tied to the facility agglomeration of urban industrial facilities, which significantly impacts population dynamics^47^. The accelerated pace of economic development and urbanization has made the influence of urban industrial facility agglomeration increasingly pronounced. This phenomenon leads to the concentration of industries in specific regions, such as the Yangtze River Delta, the Pearl River Delta, and the Beijing-Tianjin-Hebei region, resulting in distinct industrial facility agglomeration zones^25^. Moreover, the size of urban areas attracts more industrial investment and facility construction, creating a positive feedback loop between population growth and industrial development. Therefore, understanding the impact of urban industrial facility agglomeration is vital for comprehending the evolving trend of China's population distribution pattern. Building upon this understanding, this paper offers a comprehensive analysis of China's population distribution pattern changing in urban industrial facility agglomeration. The findings of this study provide a scientific basis for formulating policies that can promote regional economic development^7,8^.

#### Population spatial distribution characteristics

**Figure 2:**
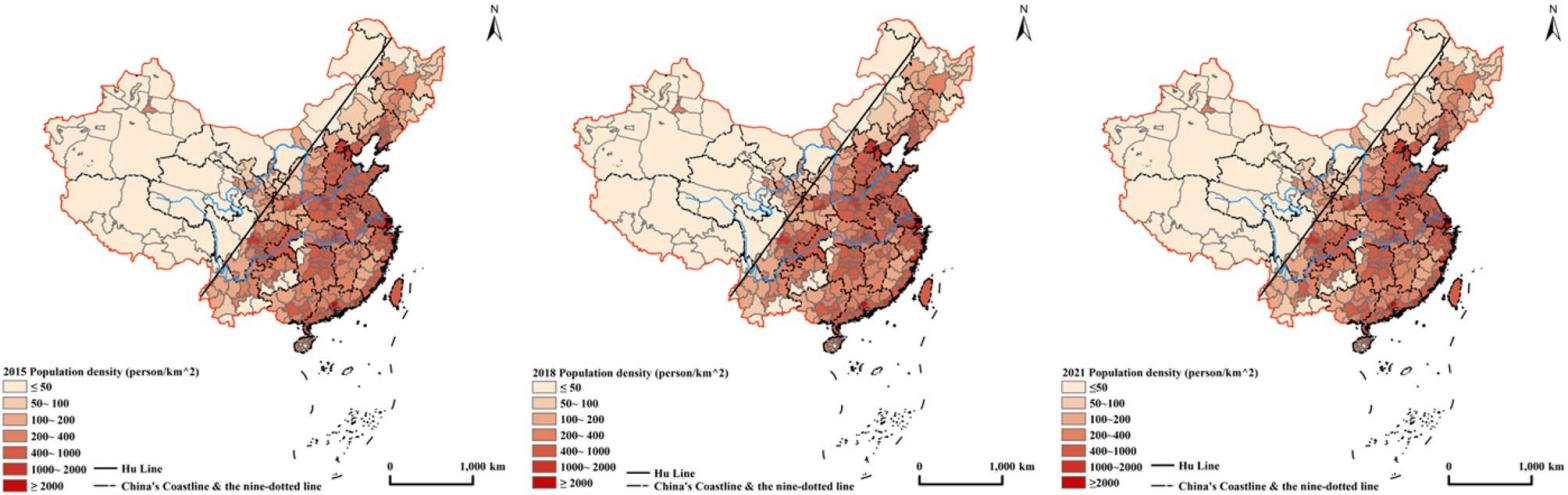
Distribution pattern of population density in China from 2015 to 2021. This map displays the distribution pattern of population density in China from 2015 to 2021. Generated using ArcGIS Desktop 10.8 (ArcMap 10.8, Release Version 10.8, Product Version 10.7.0.10450).

The Hu Line has played a crucial role in identifying China's population distribution pattern, dividing the country into the southeast and northwest regions. This line serves as a boundary, revealing significant disparities in population distribution. The densely populated areas are primarily located in the east, while the west remains sparsely populated (Fig. 2). Over the years, China has witnessed substantial population migration, predominantly occurring in the southeast. Meanwhile, the northwest has experienced higher population growth due to higher birth rates and lower emigration rates. The proportion of the population in the northwest has gradually increased from 5.77% in 1982 to 6.5% in 2020. The seventh national population census in 2020 confirms the influence of natural geographical environments on population distribution stability. From 2015 to 2021, areas with a population density of over 1000 person/km^2^ were predominantly found in the southeast, while only Lanzhou, Yinchuan, and Shihezi in the northwest had a density of over 300 person/km^2^.

The southeast side of the Hu Line, encompassing coastal areas such as Guangdong, Zhejiang, and Jiangsu, exhibits higher population density. In contrast, the northwest side, including Tibet, Xinjiang, and Qinghai, has a lower population density due to climatic and natural conditions. The eastern coastal areas have experienced higher urbanization rates, reflecting the process of population migration from rural to urban areas. The eastern region's higher urbanization rate has contributed to its relatively higher economic and social development. However, from 2015 to 2021, the aging population issue worsened, particularly in the eastern region, due to declining fertility rates and increasing life expectancy^48^. Population mobility remains high in the western region, driven by disparities in economic development between the western and eastern coastal areas^49^. Many young individuals from the western region seek better opportunities in the eastern coastal cities, resulting in a continuous decrease in the western region's population.

From 2015 to 2021, China experienced continued large-scale population migration, with some notable shifts compared to previous years. Urbanization, rising production costs, stricter management policies for migrant workers, and evolving economic and demographic structures have influenced population migration patterns. These changes have affected the prevalence of rural migrant workers, prompting some to return to their hometowns or seek opportunities elsewhere. In certain areas, stricter management policies have constrained population mobility. Furthermore, rapid economic growth and urbanization have led to aging populations and declining population figures in some cities. Conversely, economic development in the western regions has attracted population inflows. These factors collectively contribute to the transformation of population migration patterns.

#### Characteristics of population distribution equilibrium changes

**Figure 3:**
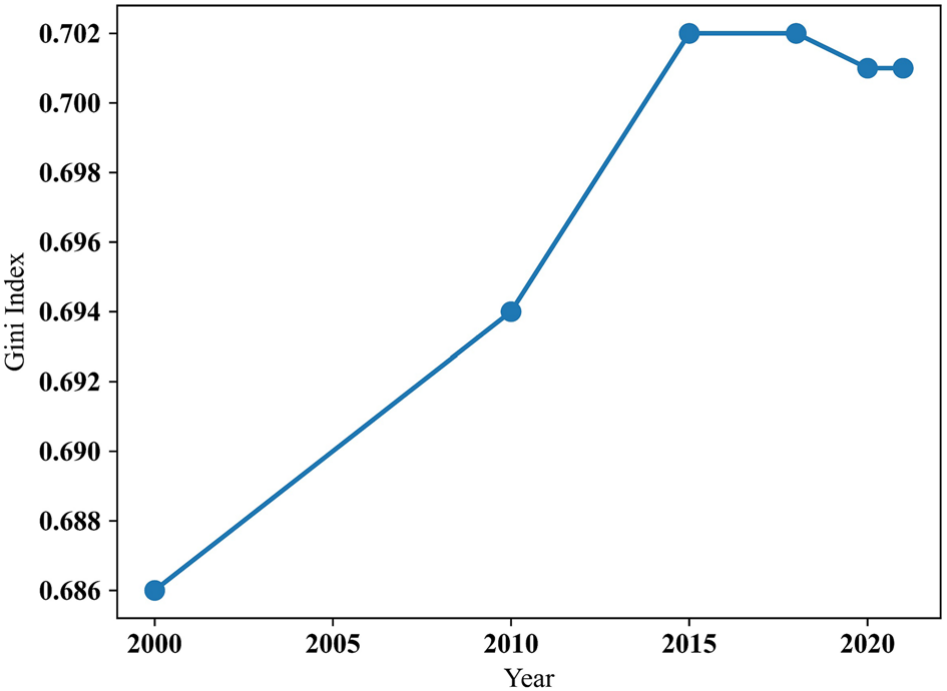
Gini coefficient of population distribution in China from 2000 to 2021.

The Gini coefficient is a practical indicator widely used to assess population distribution inequality in China. This study utilizes rasterized population data from 2000 to 2021 to calculate the Gini coefficient, revealing minor fluctuations but an overall upward trend (Fig. 3). This indicates a growing imbalance in population distribution. Efforts to foster balanced distribution include developing the Western regions' economy, improving infrastructure connectivity, and encouraging regional coordinated development. Addressing this imbalance requires persistent implementation of effective policies.

Regional disparities in economic development have intensified population distribution imbalances. Rapid economic growth in the eastern regions attracted migrants while lagging development in the west led to population outflows. Variations in natural geographical conditions also contribute to these imbalances, with the east offering favorable conditions and the west experiencing lower population densities due to limiting factors.

Analyzing specific periods, the Gini coefficient increased between 2000 and 2010 due to rapid economic development and urbanization. Between 2010 and 2015, the growth slowed as the government supported western and central-western regions, curbing inequality. From 2015 to 2018, stability was observed due to regional development efforts. Between 2018 and 2021, a slight decrease occurred as the government focused on coordinated development, and inland cities and western regions experienced growth.

The Gini coefficient ranges from 0 to 1, with 0 indicating equitable distribution and 1 representing uneven distribution. The Lorenz curve visually represents distributional inequality, with the Gini coefficient derived from it. China's Lorenz curve for population distribution demonstrates significant inequality driven by economic development, urbanization, and migration. The curve shows concentration in economically developed eastern coastal regions and large cities.

**Figure 4:**
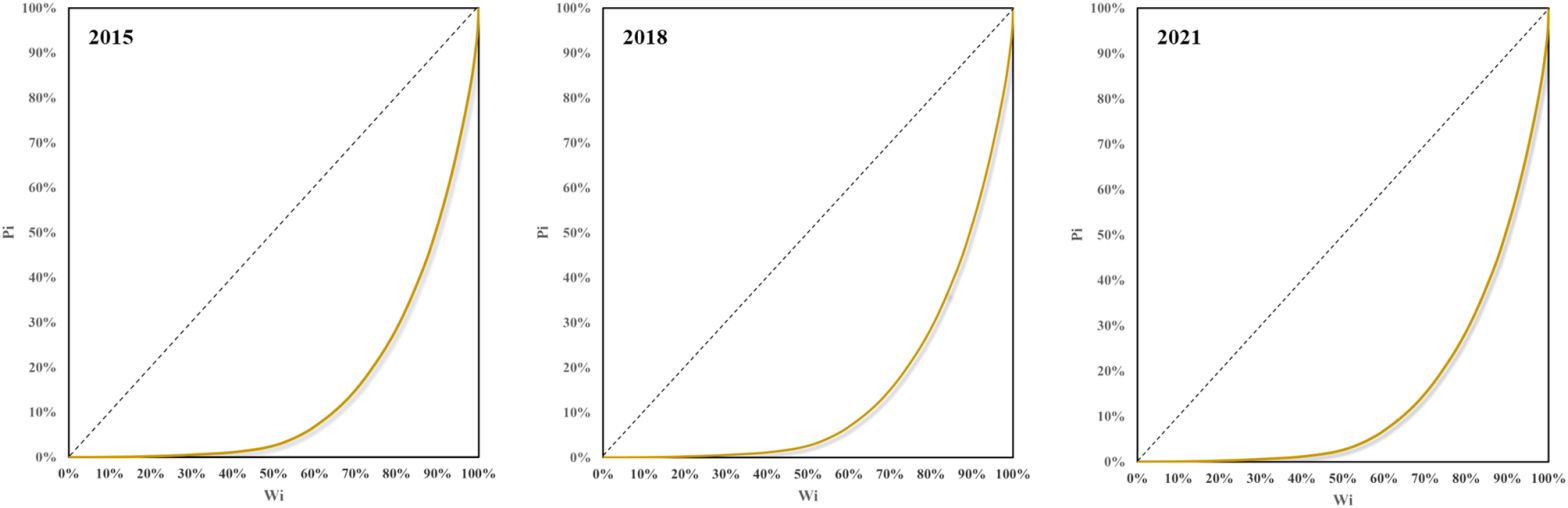
Lorenz curve of population distribution in China from 2015 to 2021.

The Gini coefficient decreased slightly by 2018 and 2021, reflecting a modest reduction in population distribution imbalance^50^. Efforts to bolster regional coordination and develop central and western regions contributed to this change. However, the imbalance persists, albeit with limited improvement. Economic transition and ongoing regional development strategies contribute to the decreasing trend^51^. The specific Lorenz curve for China's population distribution is depicted in Fig. 4.

### Spatial analysis of urban industrial facility agglomeration

Amidst globalization and urbanization, urban industrial facility agglomeration has emerged as a critical driver of economic development and urban competitiveness. Industrial facility agglomeration fosters communication and collaboration among industries, enhancing production efficiency and stimulating employment, innovation, and regional development. To uncover the intrinsic relationship between urban industrial facility agglomeration and urban population growth rates, this study utilizes spatial analysis methods to examine the influence of spatial distribution characteristics and agglomeration degrees of 12 industry types on urban population growth rates.

**Figure 5:**
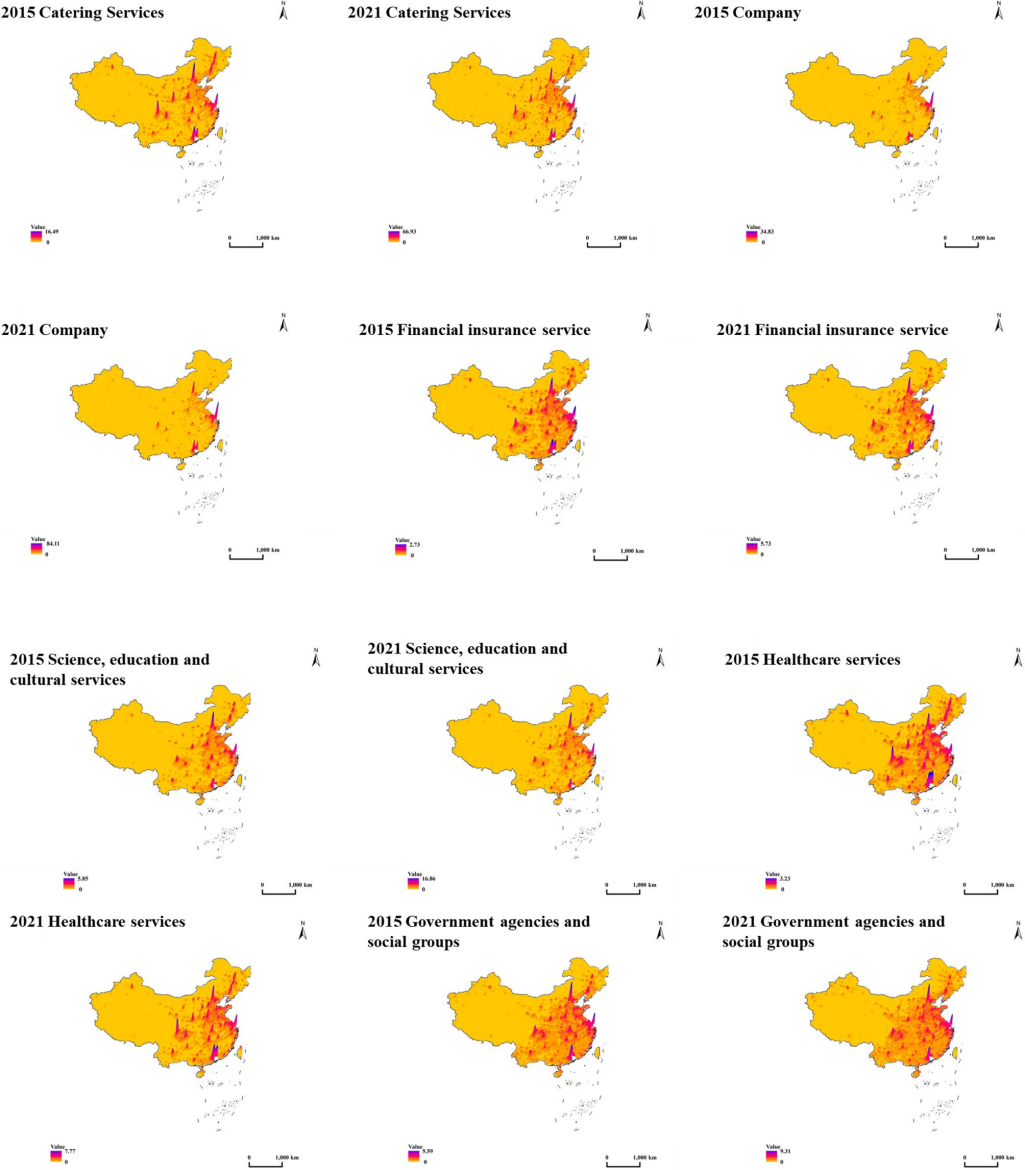
3D kernel density map of industrial facilities in each city in 2015 and 2021. A 3D kernel density map representing the spatial distribution of industrial facilities in various cities for the years 2015 and 2021. Created using ArcGIS Desktop 10.8 (ArcScene 10.8, Release Version 10.8, Product Version 10.7.0.10450).

In conjunction with POI data, this paper employs a kernel density algorithm to characterize these industry types and analyze the spatial distribution patterns of various urban industries across China, as depicted in Fig. 5. Observations from the 3D kernel density maps of the 12 industries in 2015 and 2021 reveal the following trends.

To sum up, between 2015 and 2021, the high-density regions across various industries were primarily concentrated in the Beijing-Tianjin-Hebei, Yangtze River Delta, and Pearl River Delta areas. These regions play a crucial role in China's economic development and are characterized by high output value and population density. Beijing and Shanghai consistently emerged as the top industry categories' top performers, reflecting their positions as China's political and economic hubs^52^. Furthermore, cities like Chengdu and Chongqing highly competed in specific industry sectors.

#### Global autocorrelation analysis

In this section, we utilized the Global Moran's I index to calculate the spatial autocorrelation of 12 types of industrial facilities across China in 2015, 2018, and 2021. Each industry type exhibited significant spatial autocorrelation at all three time points, indicating that industrial facilities in each region were highly correlated with adjacent geographic units. The changes in the Global Moran's I value from 2015 to 2021 are presented in Table 2.

**Table 2:** Global Moran's I value of urban industrial facilities in China from 2015 to 2021.

POI code	A	B	C	D	E	F	G	H	I	J	K	L
2021 global Moran's I	0.132	0.167	0.231	0.199	0.167	0.108	0.092	0.152	0.109	0.159	0.168	0.092
2021 z-value	9.538	12.248	17.259	14.263	12.084	8.007	7.715	11.004	8.130	11.429	12.136	6.642
2021 *p* value	0	0	0	0	0	0	0	0	0	0	0	0
2021 Var	0.000	0.000	0.000	0.000	0.000	0.000	0.000	0.000	0.000	0.000	0.000	0.000
2018 global Moran's I	0.131	0.112	0.237	0.196	0.167	0.113	0.092	0.151	0.108	0.157	0.167	0.092
2018 z-value	9.468	10.644	17.975	14.032	12.053	8.354	7.710	10.948	8.075	11.226	12.087	6.627
2018 *p* value	0	0	0	0	0	0	0	0	0	0	0	0
2018 Var	0.000	0.000	0.000	0.000	0.000	0.000	0.000	0.000	0.000	0.000	0.000	0.000
2015 global Moran's I	0.085	0.171	0.223	0.164	0.157	0.095	0.099	0.129	0.102	0.129	0.146	0.109
2015 z-value	6.266	12.798	16.802	11.780	11.325	7.086	7.440	9.442	7.552	9.308	10.591	7.824
2015 *p* value	0	0	0	0	0	0	0	0	0	0	0	0
2015 Var	0.000	0.000	0.000	0.000	0.000	0.000	0.000	0.000	0.000	0.000	0.000	0.000

The spatial autocorrelation of the 12 types of industrial facilities displayed significant spatial clustering at all three time points. Notably, the Company type had the strongest spatial correlation with Global Moran's I values of 0.223 (in 2015), 0.237 (in 2018), and 0.231 (in 2021). This suggests that the spatial distribution of Company type industrial facilities exhibited the strongest correlation across China during the study period. Additionally, Famous tourist sites, Shopping service, and Financial insurance service types also exhibited relatively high Global Moran's I value of 0.167 (in 2021), 0.199 (in 2021), and 0.167 (in 2021), respectively. The z-values of all industries at the three-time points were significantly greater than zero, and the p-values were zero, indicating that the Global Moran's I was highly significant, and spatial autocorrelation was significant across these industries^53^. These results demonstrate that the spatial distribution of the 12 types of industrial facilities in China exhibited a certain degree of clustering during the study period, which may be attributed to the economic development level, industrial structure, and policy orientation of different regions in China^54^.

By comparing the Global Moran's I value of 12 industries across China in 2015, 2018, and 2021, this article can analyze the changing trends in spatial autocorrelation. Catering Services showed a consistent year-on-year increase at all three time points, indicating a continuous rise in the spatial agglomeration of catering facilities. From 2015 to 2018, Famous tourist sites displayed a decreasing trend but slightly rebounded from 2018 to 2021, suggesting a fluctuation in the spatial agglomeration of tourist attractions yet remaining relatively stable overall. The Global Moran's I value of Company facilities increased from 2015 to 2021, indicating an overall increase in the spatial agglomeration of company facilities^55^. Similarly, Shopping service, Financial insurance service, Science, education and cultural services, Domestic services, Sports and Leisure Services, Healthcare services, and Government agencies and social groups all showed an increasing trend in their Global Moran's I value from 2015 to 2021, indicating an overall increase in the spatial agglomeration of facilities in these industries^56^. However, Business residence showed an overall decreasing trend from 2015 to 2021, indicating a reduction in the spatial agglomeration of commercial and residential facilities. Accommodation services also decreased from 2015 to 2021, indicating decreased spatial agglomeration of accommodation facilities.

#### Local autocorrelation analysis

**Figure 6:**
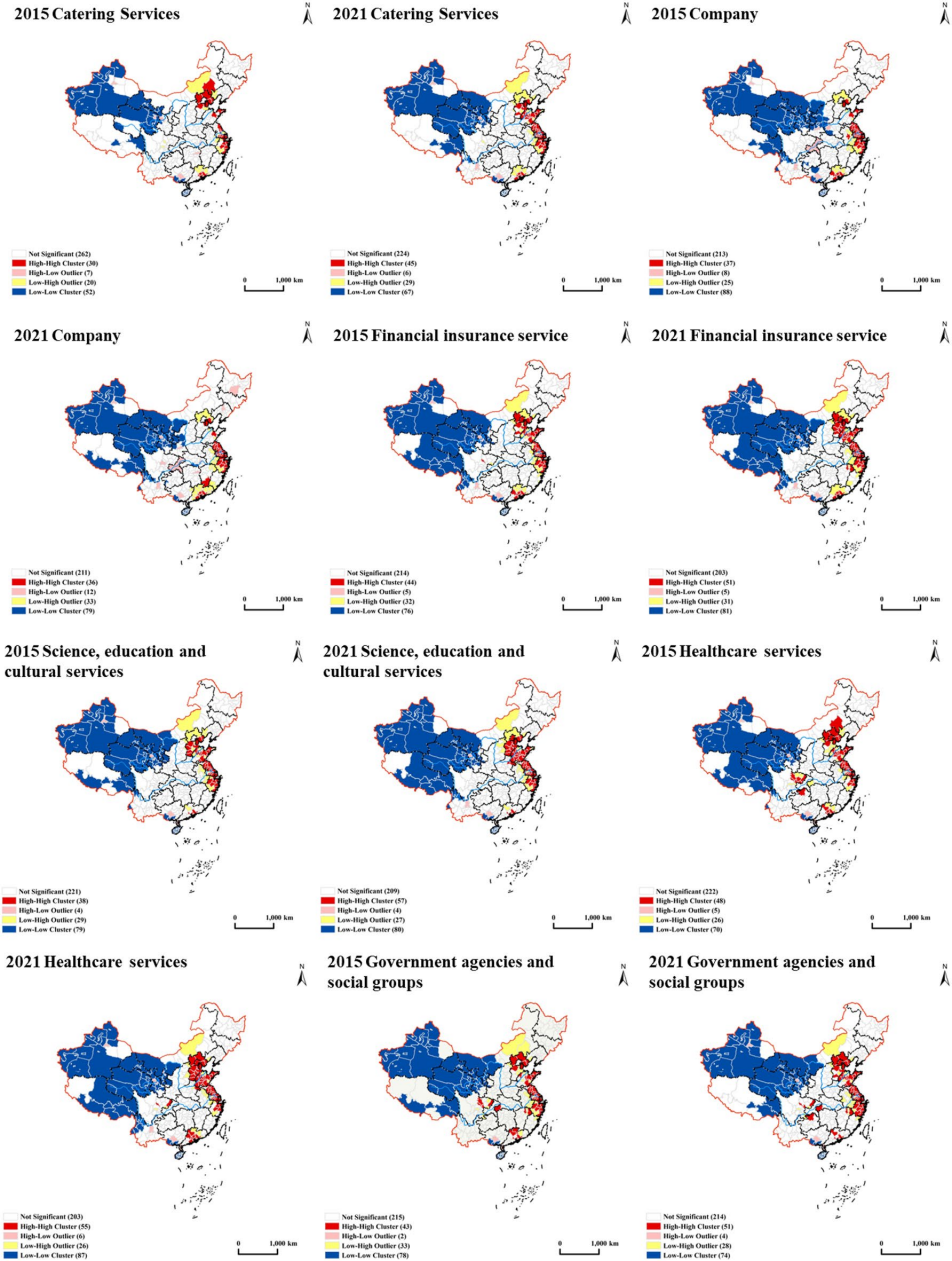
LISA map of industrial facility agglomeration in China in 2015 and 2021. This map visualizes the Local Indicators of Spatial Association for the agglomeration of industrial facilities in China during 2015 and 2021. Produced with ArcGIS Desktop 10.8 (ArcMap 10.8, Release Version 10.8, Product Version 10.7.0.10450).

This section uses the Local Moran's I index to compute the spatial autocorrelation of 12 industrial facilities across China and examine the interrelationships among various industry types among administrative units at or above the prefectural level. The LISA map is an indicator that assesses the degree of similarity and dissimilarity and its significance between spatial unit attributes and neighboring units. LISA clustering maps depict four distinct types of spatial autocorrelation relationships: High-High (H–H), Low–Low (L–L), Low–High (L–H), and High–Low (H–L). H–H type represents a high level of industrial facility agglomeration in the study area and its adjacent regions. In contrast, the L–L type indicates a low level of industrial facility agglomeration in the study area and its surrounding areas. The L–H type suggests that the industrial facility agglomeration level is low in the study area but high in its surrounding areas. In contrast, the H–L type indicates a high industrial facility agglomeration level in the study area but low in its surrounding areas. By calculating the Moran's I value of industrial facility agglomeration distribution among administrative units at or above the prefectural level in 2015 and 2021 and drawing LISA clustering maps based on Z-test values (*P* = 0.05), readers can observe the changes and trends of industrial facility agglomeration as shown in Fig. 6. An in-depth analysis of data for the 12 types of industries reveals that the number of prefectural and higher-level units for each type of industry generally exhibits an increasing trend. Moreover, the Beijing-Tianjin-Hebei, the Yangtze River Delta, and the Pearl River Delta regions show high agglomeration levels in most industries. However, regions such as Tibet, Qinghai, Xinjiang, Gansu, southwest Inner Mongolia, southern Guangxi, and Hainan exhibit relatively low agglomeration levels in most industries. Further research into the factors that affect the spatial agglomeration of facilities in various industries, such as policy changes, regional development strategies, industrial structure adjustments, and population mobility, can provide targeted recommendations for urban planning, industrial policy development, and regional economic development.

In conclusion, the spatial autocorrelation relationships of various industries underwent significant changes between 2015 and 2021. In general, the number of H–H level prefectural and higher-level administrative units increased in the Beijing-Tianjin-Hebei, Yangtze River Delta, and Pearl River Delta regions, indicating an increase in agglomeration. Conversely, the number of H–L level units is relatively small and mainly concentrated in central and northeastern China's provincial capitals or central cities. The distribution of L–H level units is uneven among industries, primarily concentrated in the northern part of Hebei and southeastern Anhui. In contrast, the number of L–L level units is relatively high and mainly distributed in areas with lower economic development levels, such as Tibet, Qinghai, and Xinjiang. A comparative analysis of the 2015 and 2021 data shows that industry facility agglomeration tends to concentrate, particularly in economically developed regions. This trend is closely linked to policy support, infrastructure construction, and market demand. However, some relatively underdeveloped regions still face challenges in industrial development. Therefore, more support is necessary for these areas in the future to promote industry development and spatial optimization.

### Spatial heterogeneity analysis of population distribution and industrial facility agglomeration

This study used the geographical detectors method to analyze the agglomeration of various industries and its impact on urban population growth rates. Table 3 presents the results of the factor detector for 2015 and 2021, including q-statistic values and *p* values. The findings reveal significant disparities in the influence of different factors on population growth rates between the two periods. Note that “A B C …” represents different types of POIs (A, B, C, …, L).

**Table 3:** 2015 and 2021 factor detector results.

Year	POI code	A	B	C	D	E	F	G	H	I	J	K	L
2015	qstatistic	0.170	0.155	0.150	0.161	0.171	0.217	0.183	0.162	0.165	0.106	0.137	0.123
	*p* value	0.000	0.000	0.000	0.000	0.000	0.000	0.000	0.000	0.000	0.005	0.000	0.002
2021	qstatistic	0.063	0.075	0.064	0.064	0.055	0.092	0.042	0.055	0.053	0.068	0.096	0.038
	*p* value	0.022	0.022	0.022	0.034	0.045	0.000	0.150	0.056	0.051	0.008	0.000	0.251

In 2015, Science, Education, and Cultural Services (F), Business Residence (G), and Financial Insurance Services (E) had a substantial impact on urban population growth rates (q = 0.217, q = 0.183, and q = 0.171, respectively, all with *p* < 0.001). This suggests that these factors played a significant role in shaping population growth in 2015. However, in 2021, Science, Education, and Cultural Services (F) and Government Agencies and Social Groups (K) emerged as the key influencers (q = 0.092 and q = 0.096, respectively, both with *p* < 0.001). These findings indicate that the factors influencing population growth rates have evolved over time, with Science, Education, and Cultural Services and Government Agencies and Social Groups gaining increasing importance.

It is worth noting that certain factors had a relatively low impact on population growth rates in both periods. For example, Domestic Services (H) had a q-statistic of 0.162 in 2015, which decreased to 0.055 in 2021. Similarly, Accommodation Services (L) had a q-statistic of 0.123 in 2015, further decreasing to 0.038 in 2021. These results suggest that these factors have a minor influence on urban population growth, with varying degrees of impact across different periods.

The analysis reveals a shift in the key factors influencing urban population growth rates over time. Government Agencies and Social Groups gained significance, while factors like Business Residence and Financial Insurance Services exhibited a weaker impact. Notably, Science, Education, and Cultural Services consistently strongly influenced both periods. This emphasizes the crucial role of education, scientific research, and government policies in shaping population distribution and industry facility agglomeration dynamics.

### The evolution and influence of China's industrial facility agglomeration on the shape of population distribution pattern

**Figure 7:**
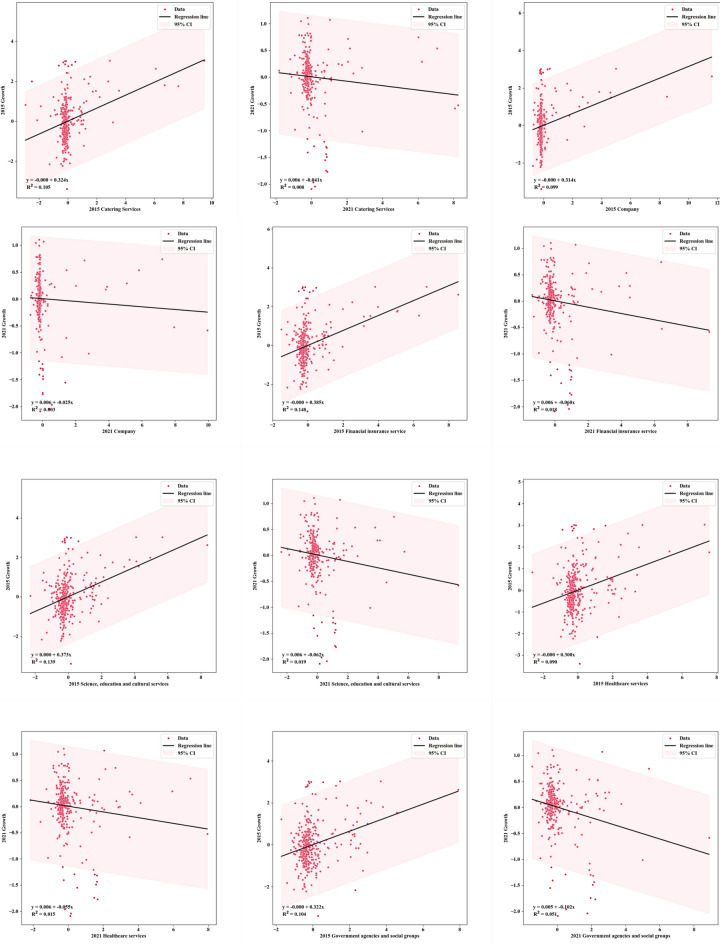
Scatter plot of China’s urban population growth rate and urban industrial facility agglomeration in 2015 and 2021.

This study examines the impact of industrial facility agglomeration on population distribution patterns in China from 2015 to 2021. Using a linear regression model and data visualization techniques, the relationships between urban industrial facilities and population growth rates are analyzed^57^. The findings reveal linear relationships between industrial facility agglomerations and population growth rates, with variations in strength and direction between the two time periods (Fig. 7). Please note that the Population Growth Rate is calculated as follows: (End-of-Year Population − Beginning-of-Year Population)/Beginning-of-Year Population × 100%. In 2015, Financial Insurance Services had a significant impact on population growth rates. However, this influence weakened in 2021 due to the deceleration of overall population growth and the emergence of other influential factors. The correlation coefficients between industrial facility agglomerations and population growth rates shifted from positive to negative during this period, reflecting changes in China's population policies and economic structure^58^. Notably, the relationships between Famous Tourist Sites, Science, Education and Cultural Services, Government Agencies and Social Groups, and population growth rates weakened in 2021, attributed to government initiatives promoting industrial transformation and upgrading^59^. Emerging industries, high-tech, and service sectors have gained more attractiveness, while traditional industrial facilities have comparatively diminished appeal.

**Figure 8:**
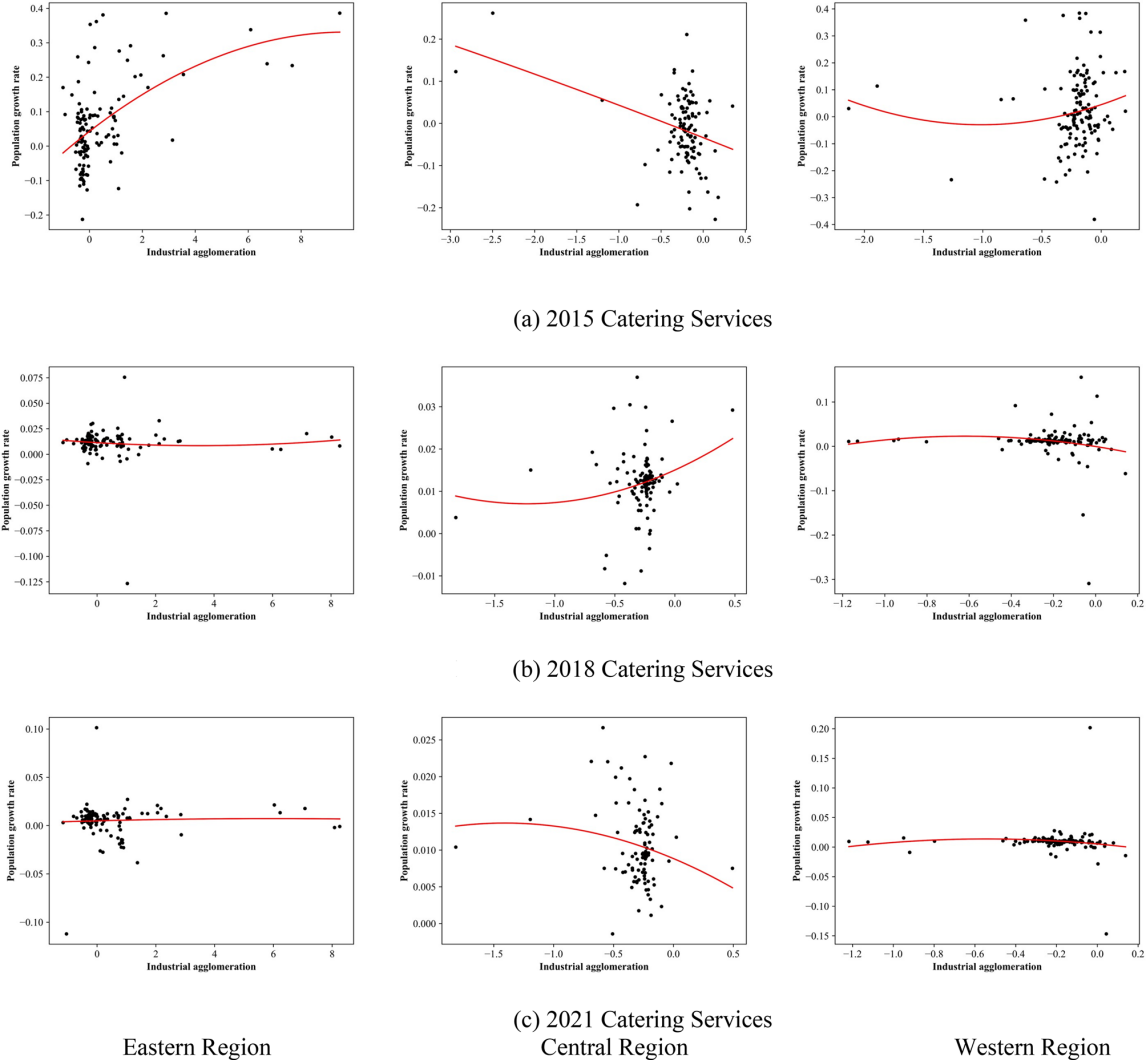
The relationship between industrial facility agglomeration and population growth by dividing China into eastern region, central region, and western region.

This analysis examines the relationship between urban industrial facility agglomeration and urban population growth in different regions of China. The focus is on the Catering Services industry as a case study (Fig. 8), with supplementary materials providing information on other industries.

In 2015, the eastern region showed high industrial facility agglomeration in Catering Services, positively correlated with population growth. The central region had lower industrial facility agglomeration, negatively correlated with population growth. The western region had low industrial facility agglomeration, with an insignificant correlation to population growth. By 2018, the eastern region maintained high industrial facility agglomeration in Catering Services, but the correlation with population growth was unclear. The central region showed low industrial facility agglomeration, positively correlated with population growth. The western region maintained low industrial facility agglomeration, with an insignificant correlation to population growth. In 2021, the eastern region still had high industrial facility agglomeration in Catering Services, but the relationship with population growth was unclear. The central region had low industrial facility agglomeration, negatively correlated with population growth. The western region had low industrial facility agglomeration, with an insignificant correlation to population growth.

Overall, industrial facility agglomeration for other industries had specific characteristics. In the eastern region, agglomeration concentrated between (0, 2), while in the central and western regions, it concentrated between (− 0.5, 0). Various factors influenced these patterns:

*Eastern Region* Rapid economic growth and urbanization led to industry saturation, resulting in small changes in agglomeration. Urbanization and population aging slowed population growth and weakened the correlation with agglomeration. Economic restructuring towards the service and innovation sectors is crucial for sustainable growth.

*Central Region* Lower economic development and social resources limited population growth compared to the eastern region. Attracting advantageous industries from the east can improve employment and economic development. Industries like Company, Science, Education, Business Residence, and Accommodation Services performed significantly.

*Western Region* Initially reliant on natural resource development, the region faced limitations and weakened agglomeration. Strengthening industrial diversification and fostering innovation-driven development is necessary. Industries like Famous Tourist Sites, Company, Financial Insurance Services, and Science, Education and Cultural Services performed remarkably. Focus on tourism, business support, financial services, and science, education, and culture investments can promote growth.

This study conducted a multiple regression analysis on the relationship between urban population growth rates and agglomerations of industrial facilities in China. Data from 2015, 2018, and 2021 were analyzed, and comprehensive results can be found in Table 4.

**Table 4:** Multiple regression results of population growth rate and agglomeration degree of various industrial facilities.

Years	Multiple regression model
2015	$Growth = 0.689E_{IA} - 0.172G_{IA} - 0.193L_{IA} + \varepsilon_{it}$ $R^{2} = 166 P < 0.01 VIF_{{E_{IA} }} = 4.25 VIF_{{G_{IA} }} = 1.89 VIF_{{L_{IA} }} = 6.89$
2018	$$Growth = - 0.188C_{IA} + 0.294E_{IA} - 0.251F_{IA} - 2.369$$ $$R^{2} = 0.047 P < 0.01 VIF_{{C_{IA} }} = 2.00 VIF_{{E_{IA} }} = 1.89 VIF_{{F_{IA} }} = 3.65 $$
2021	$$Growth = 0.541A_{IA} + 0.614C_{IA} - 0.273G_{IA} - 1.261H_{IA} + 0.566K_{IA} + 0.254$$ $$R^{2} = 0.053 P < 0.01 VIF_{{A_{IA} }} = 1.14 VIF_{{C_{IA} }} = 6.89 VIF_{{G_{IA} }} = 2.49 $$ $$ VIF_{{H_{IA} }} = 1.68 VIF_{{K_{IA} }} = 1.13$$

In 2015, after controlling for other factors, Financial Insurance Services agglomeration showed a significantly positive correlation with urban population growth rates. On the other hand, Business Residence and Accommodation Services agglomerations had significantly negative correlations. These findings suggest that Financial Insurance Services were attractive to urban populations in 2015, while Business Residence and Accommodation Services were less appealing. The regression model explained about 16.6% of the variation in population growth rates (R^2^ = 0.166).

In 2018, the regression results showed a significantly negative correlation between Company agglomeration and urban population growth rates. Financial Insurance Services and Science, Education, and Cultural Services agglomerations had significant positive and negative correlations. This indicates that Financial Insurance Services remained attractive to urban populations in 2018, while Companies and Science, Education, and Cultural Services were less appealing. The regression model explained about 4.7% of the variation in population growth rates (R^2^ = 0.047).

In 2021, Catering Services, Companies, Government Agencies, and Social Groups agglomerations were significantly correlated with urban population growth rates. Business Residence and Domestic Services agglomerations had significantly negative correlations. This suggests that Catering Services, Companies, Government Agencies, and Social Groups were highly appealing to urban populations in 2021, while Business Residence and Domestic Services were less enticing. The regression model explained about 5.3% of the variation in population growth rates (R^2^ = 0.053).

Over the study period, the relationships between population growth rates and agglomerations of industrial facilities in China's administrative units experienced notable changes. Financial Insurance Services consistently attracted urban populations, while the appeal of other industries varied. This highlights the impact of industrial restructuring and transformation on population distribution patterns during China's urban development. As China's economy advances and its industrial structure evolves, high-value-added industries and service sectors will be crucial in shaping future urban population distribution. Therefore, the government must prioritize optimizing industrial facility layouts, promoting industrial restructuring and transformation, and fostering sustainable urban development to address these evolving population patterns.

## Discussion

The findings of this study offer unique insights into the intricate relationship between urban industrial facility agglomeration and population growth in China. By leveraging the LandScan population dataset and the POI spatial dataset, along with mature spatial analysis techniques and multiple regression models, this study breaks new ground in understanding the dynamics of population distribution and industrial facility agglomeration.

One of the distinctive contributions of this study lies in its examination of the evolving factors influencing urban population growth rates. While previous studies have focused on traditional determinants, this research highlights the increasing significance of Government Agencies and Social Groups alongside the consistent impact of Science, Education, and Cultural Services.

Furthermore, this study unveils the shifting correlations between industrial facility agglomerations and population growth rates. The linear relationships observed in 2015 and 2021, albeit with variations in strength and direction, highlight the nuanced nature of these connections. This demonstrates that many factors influence population growth in China, and industrial facility agglomeration is just one piece of the puzzle. These findings challenge conventional assumptions and underscore the need for a comprehensive understanding of the complex interplay between industrial facility agglomeration, population dynamics, and other contextual factors.

The regional disparities in industrial facility agglomeration patterns identified in this study further underscore its significance. The unique characteristics and development trajectories of China's eastern, central, and western regions call for tailored strategies to optimize industrial structures, foster innovation-driven industries, and promote sustainable population growth. This regional approach to urban development is crucial for addressing the diverse challenges and harnessing the opportunities presented by China's evolving economic landscape.

Moreover, the innovative use of the LandScan population and POI spatial datasets sets this study apart. These datasets provide comprehensive, real-time, and fine-grained information, enabling a more nuanced understanding of population distribution and industrial facility agglomeration dynamics. The integration of mature spatial analysis techniques and multiple regression models enhances the findings' accuracy and reliability, contributing to this research's methodological innovation.

While the study offers valuable insights, it acknowledges certain limitations that future research could address. For a more comprehensive prediction and explanation of population growth rates, future studies should consider additional factors like infrastructure, the policy environment, social welfare, and educational resources^60^. It would also be beneficial to examine the interplay among different industrial facility agglomerations and assess their collective impact on urban development and population growth. Furthermore, analyzing the effects of various city types and regional disparities on the relationship between industrial facility agglomeration and population growth rates could shed light on spatial heterogeneity in population distribution and industrial facility agglomeration^61^. Additionally, future research could explore potential nonlinear relationships using nonlinear or multivariate regression models^62^.

## Conclusion

This study employs the LandScan population dataset and the POI spatial dataset, in conjunction with a range of population spatial analysis techniques and multiple regression models, to examine the interplay between population distribution patterns and industrial facility agglomeration in 352 Chinese administrative units at the prefecture level and above. The primary findings are as follows:

1. Factors influencing urban population growth rates varied between 2015 and 2021, with Government Agencies and Social Groups gaining significance. Science, Education, and Cultural Services consistently influenced population growth, highlighting the importance of education, research, and government policies in the relationship between population distribution and industrial facility agglomeration.
2. The correlation between industrial facility agglomerations and urban population growth rates exhibited linear relationships in 2015 and 2021, but the strength and direction of these relationships differed. Correlation coefficients shifted from positive to negative, indicating changes in China's population growth rate and the influence of other factors.
3. Industrial facility agglomeration patterns varied across regions, with the eastern region experiencing rapid economic growth and urbanization, resulting in saturated industries. The central region faced economic development and resource limitations, while the western region relied on natural resources but required diversification. The focus should be optimizing industrial structure, fostering innovation-driven industries, and developing service sectors to achieve sustainable population growth and economic development^63^.

Based on the study's findings, the following specific development strategies and policy recommendations are proposed for local governments and planners:

1. *Promote the transition to high-value-added sectors* Encourage shifting from traditional manufacturing industries to sectors with higher value and innovation potential, such as technology, research and development, and knowledge-intensive services. This can be achieved by providing targeted investments, incentives, and supportive policies that facilitate the growth and development of innovation-driven enterprises. Emphasizing a diversified and technologically advanced industrial landscape will create employment opportunities, attract skilled workers, and drive sustainable population growth.
2. *Address regional disparities* Tailor strategies to each region's unique characteristics and challenges. In the eastern region, focus on optimizing the existing industrial structure and transitioning towards service-oriented and innovation-driven sectors. The central region should attract advantageous industries from the eastern region, expand employment opportunities, and enhance economic development. In the western region, diversify the industrial base, reduce dependence on natural resources, and promote emerging industries. These approaches will foster balanced regional development and sustainable population growth.
3. *Establish a harmonious relationship between industrial facility agglomeration and population growth* Prioritize measures that optimize the layout of industrial facilities to enhance efficiency and minimize environmental impacts. Support green and sustainable urban planning practices, including providing quality public services, infrastructure development, and creating livable urban environments. Increase investments in education, research, and cultural sectors to promote human capital development and attract talent. Strengthen social welfare systems to address income disparities and improve urban residents' overall quality of life. These efforts will ensure that population growth is accompanied by enhanced livability and improved well-being.

Previous research on population distribution and industrial facility agglomeration in China has emphasized economic factors as the primary drivers of urban population growth. These studies often established a stable positive correlation between industrial facility agglomerations and population growth rates, attributing urbanization and economic development as the key factors. While this study aligns with this fundamental understanding, it provides significant contributions and novel insights that differentiate it from existing research:

1. *Evolving Nature of the Relationship* Existing studies have generally treated the relationship between industrial facility agglomeration and population growth as relatively static. In contrast, this research reveals a dynamic and evolving relationship. We demonstrate that the influence of industrial agglomerations on population growth rates has shifted over the study period, moving from a predominantly positive correlation to a more complex and nuanced interaction.
2. *Changing Role of Non-Economic Factors* Previous research often neglected or downplayed the role of non-economic factors, such as government policies and social dynamics, in shaping population distribution patterns. This study, however, highlights the increasing significance of these factors. Government Agencies and Social Groups have emerged as influential determinants of urban population growth, indicating a shift in the landscape of influence away from purely economic considerations.
3. *Necessity for Tailored Regional Strategies* Prior studies have recognized regional disparities in population distribution and industrial agglomeration, but this research underscores the need for region-specific strategies. This article provides concrete evidence of how different regions within China exhibit varying patterns of industrial facility agglomeration, necessitating tailored approaches. This regional differentiation emphasizes that a one-size-fits-all policy approach may not effectively address each region's unique challenges and opportunities.
4. *Emphasis on Diversified, Innovation-Driven Industries* A notable distinction in this study is the emphasis on the need for diversified, innovation-driven industries to foster sustainable population growth. Previous research tended to focus primarily on traditional manufacturing industries. We advocate for transitioning to high-value-added sectors, such as technology, research and development, and knowledge-intensive services, aligning with global trends toward knowledge-based economies.

The differences between this study and previous research can be attributed to several factors. Firstly, from 2015 to 2021, the study period captures a period of rapid socio-economic transformation in China, leading to shifts in population dynamics and industrial structures. Additionally, this research's comprehensive dataset and advanced spatial analysis techniques enable a more nuanced understanding of the interplay between factors. Lastly, this study emphasizes interdisciplinary perspectives, integrating economic, social, and policy dimensions to offer a holistic view of the subject.

In conclusion, this study underscores the dynamic interplay between urban industrial agglomeration and population growth in China, revealing the evolving nature of the underlying factors. The influence of government agencies, social groups, and sectors such as science, education, and cultural services has grown over time. The correlations between industrial agglomeration and population growth rates have exhibited notable shifts in intensity and direction, mirroring changes in China's overall population growth rate and the escalating impact of additional determinants. Furthermore, regional disparities in industrial agglomeration patterns underscore the necessity for tailored strategies, optimizing industrial structures, cultivating innovation-driven sectors, and fostering sustainable population growth in different regions. As China advances its economy and continues industrial restructuring, policymakers must accord priority to reconfiguring industrial facility layouts, facilitating industrial transformation, and propelling sustainable urban development to accommodate the ever-evolving patterns of population distribution. By implementing these measures, China can effectively navigate the complexities of urbanization and establish a harmonious relationship between industrial agglomeration and population growth in the future.

### Supplementary Information

Supplementary Information 1.

Supplementary Information 2.

Supplementary Information 3.

Supplementary Information 4.

Supplementary Information 5.

Supplementary Information 6.

Supplementary Information 7.

Supplementary Information 8.

## Data Availability

The datasets used and/or analysed during the current study available from the corresponding author on reasonable request.

## References

[1] Jarzebski MP, Elmqvist T, Gasparatos A, Fukushi K, Eckersten S, Haase D, Goodness J, Khoshkar S, Saito O, Takeuchi K, Theorell T, Pu J (2021). Ageing and population shrinking: Implications for sustainability in the urban century. NPJ Urban Sustain..

[2] Folke C, Polasky S, Rockström J, Galaz V, Westley F, Lamont M, Scheffer M, Österblom H, Carpenter SR, Chapin FS, Seto KC, Walker BH (2021). Our future in the Anthropocene biosphere. Ambio.

[3] Ye J, Chen Z, Peng B (2021). Is the demographic dividend diminishing in China? Evidence from population aging and economic growth during 1990–2015. Rev. Dev. Econ..

[4] Zhang X, Guo F, Zhai Z (2019). China’s demographic future under the new two-child policy. Popul. Res. Policy Rev..

[5] Liang L, Wang Z, Li J (2019). The effect of urbanization on environmental pollution in rapidly developing urban agglomerations. J. Clean. Prod..

[6] Qiao L, Li Y, Liu Y, Yang R (2016). The spatio-temporal change of China's net floating population at county scale from 2000 to 2010. Asia Pac. Viewpoint.

[7] Chen M, Gong Y, Li Y, Lu D, Zhang H (2016). Population distribution and urbanization on both sides of the Hu Huanyong Line: Answering the Premier’s question. J. Geogr. Sci..

[8] Chen M, Liu W, Lu D (2016). Challenges and the way forward in China’s new-type urbanization. Land Use Policy.

[9] Xi Y, Qiang L, Zhengdong H, Renzhong G (2022). Characterising population spatial structure change in Chinese cities. Cities.

[10] Deng X, Yu M (2021). Does the marginal child increase household debt?—Evidence from the new fertility policy in China. Int. Rev. Financ. Anal..

[11] Yang Q, He L (2017). Spatiotemporal changes in population distribution and socioeconomic development in China from 1950 to 2010. Arab. J. Geosci..

[12] Zaborovskaia O, Nadezhina O, Avduevskaya E (2020). The impact of digitalization on the formation of human capital at the regional level. J. Open Innov. Technol. Market Complex..

[13] Li W, Zhang Y, Yang C, Gong W, Wang C, Zhang R (2022). Does producer services agglomeration improve urban green development performance of the Yangtze River Economic Belt in China?. Ecol. Ind..

[14] Liu K, Liu X, Long H, Wang D, Zhang G (2022). Spatial agglomeration and energy efficiency: Evidence from China's manufacturing enterprises. J. Clean. Prod..

[15] Marshall A (1890). Principles of Economics.

[16] Weber A (1962). Theory of the Location of Industries.

[17] Krugman P (1991). Increasing returns and economic geography. J. Political Econ..

[18] Nielsen BB, Asmussen CG, Weatherall CD, Lyngemark DH (2021). Marshall vs Jacobs agglomeration and the micro-location of foreign and domestic firms. Cities.

[19] Liao B, Li L (2022). Spatial division of labor, specialization of green technology innovation process and urban coordinated green development: Evidence from China. Sustain. Cities Soc..

[20] Huang D, Liu Z, Zhao X, Zhao P (2017). Emerging polycentric megacity in China: An examination of employment subcenters and their influence on population distribution in Beijing. Cities.

[21] Chen Q, Guan X, Huan TC (2021). The spatial agglomeration productivity premium of hotel and catering enterprises. Cities.

[22] Lan T, Shao G, Xu Z, Tang L, Sun L (2021). Measuring urban compactness based on functional characterization and human activity intensity by integrating multiple geospatial data sources. Ecol. Ind..

[23] Gao J, Song G, Sun X (2020). Does labor migration affect rural land transfer? Evidence from China. Land Use Policy.

[24] Wang X, Wang Q (2021). Research on the impact of green finance on the upgrading of China's regional industrial structure from the perspective of sustainable development. Resour. Policy.

[25] Wang W, Gong J, Wang Y, Shen Y (2021). Exploring the effects of rural site conditions and household livelihood capitals on agricultural land transfers in China. Land Use Policy.

[26] Ning L, Wang F, Li J (2016). Urban innovation, regional externalities of foreign direct investment and industrial agglomeration: Evidence from Chinese cities. Res. Policy.

[27] Yang Z, Song T, Chahine T (2016). Spatial representations and policy implications of industrial co-agglomerations, a case study of Beijing. Habitat Int..

[28] Henderson JV (2003). Marshall's scale economies. J. Urban Econ..

[29] Lin HL, Li HY, Yang CH (2011). Agglomeration and productivity: Firm-level evidence from China's textile industry. China Econ. Rev..

[30] Lu C, Pang M, Zhang Y, Li H, Lu C, Tang X, Cheng W (2020). Mapping urban spatial structure based on poi (point of interest) data: A case study of the central city of Lanzhou, China. ISPRS Int. J. Geo-Inf..

[31] Yu Z, Liu X (2021). Urban agglomeration economies and their relationships to built environment and socio-demographic characteristics in Hong Kong. Habitat Int..

[32] Tonne C, Adair L, Adlakha D, Anguelovski I, Belesova K, Berger M, Brelsford C, Dadvand P, Dimitrova A, Giles-Corti B, Heinz A, Adli M (2021). Defining pathways to healthy sustainable urban development. Environ. Int..

[33] Lang W, Chen T, Li X (2016). A new style of urbanization in China: Transformation of urban rural communities. Habitat Int..

[34] Wang L, Xue C (2019). Spatio-temporal characteristics and influencing factors of urban floating population in China from 2011 to 2015. Chin. J. Popul. Resour. Environ..

[35] Chen X, Chang CP (2020). Fiscal decentralization, environmental regulation, and pollution: a spatial investigation. Environ. Sci. Pollut. Res..

[36] Hou M, Deng Y, Yao S (2022). Coordinated relationship between urbanization and grain production in China: Degree measurement, spatial differentiation and its factors detection. J. Clean. Prod..

[37] Zhang J, Zhang K, Zhao F (2020). Research on the regional spatial effects of green development and environmental governance in China based on a spatial autocorrelation model. Struct. Change Econ. Dyn..

[38] Bisello A, Antoniucci V, Marella G (2020). Measuring the price premium of energy efficiency: A two-step analysis in the Italian housing market. Energy Build..

[39] Liu MY, Li QH, Zhang YJ, Ma Y, Liu Y, Feng W, Hou CB, Amsalu E, Li X, Wang W, Li WM, Guo XH (2018). Spatial and temporal clustering analysis of tuberculosis in the mainland of China at the prefecture level, 2005–2015. Infect. Dis. Poverty.

[40] Gao W, Lu S, Zhang X, He Q, Huang W, Lin B (2023). Impact of 3D modeling behavior patterns on the creativity of sustainable building design through process mining. Autom. Constr..

[41] Li Z, Hu M, Wang Z (2020). The space-time evolution and driving forces of county economic growth in China from 1998 to 2015. Growth Change.

[42] Zhao R, Zhan L, Yao M, Yang L (2020). A geographically weighted regression model augmented by Geodetector analysis and principal component analysis for the spatial distribution of PM2.5. Sustain. Cities Soc..

[43] Bi H, Ye Z (2021). Exploring ridesourcing trip patterns by fusing multi-source data: A big data approach. Sustain. Cities Soc..

[44] Endri E, Sari AK, Budiasih Y, Yuliantini T, Kasmir K (2020). Determinants of profit growth in food and beverage companies in Indonesia. J. Asian Finance Econ. Bus..

[45] Manning RL (1996). Is the insurance aspect of producer liability valued by consumers? Liability changes and childhood vaccine consumption. J. Risk Uncertain..

[46] Naji HA, Xue Q, Zhu H, Li T (2021). Forecasting taxi demands using generative adversarial networks with multi-source data. Appl. Sci..

[47] Ouyang X, Xu J, Li J, Wei X, Li Y (2022). Land space optimization of urban-agriculture-ecological functions in the Changsha-Zhuzhou-Xiangtan Urban Agglomeration, China. Land Use Policy.

[48] Ren Y, Tian Y, Zhang C (2022). Investigating the mechanisms among industrial agglomeration, environmental pollution and sustainable industrial efficiency: A case study in China. Environ. Dev. Sustain..

[49] Ma F, Li J, Ma H, Sun Y (2022). Evaluation of the regional financial efciency based on SBM-Shannon entropy model. Procedia Comput. Sci..

[50] Merabet GH, Essaaidi M, Haddou MB, Qolomany B, Qadir J, Anan M, Al-Fuqaha A, Abid MR, Benhaddou D (2021). Intelligent building control systems for thermal comfort and energy-efficiency: A systematic review of artificial intelligence-assisted techniques. Renew. Sustain. Energy Rev..

[51] Yu H (2021). The Guangdong-Hong Kong-Macau greater bay area in the making: Development plan and challenges. Camb. Rev. Int. Aff..

[52] Wu Y, Wei YD, Li H, Liu M (2022). Amenity, firm agglomeration, and local creativity of producer services in Shanghai. Cities.

[53] Su J, Yin H, Kong F (2021). Ecological networks in response to climate change and the human footprint in the Yangtze River Delta urban agglomeration, China. Landsc. Ecol..

[54] Escudero V, Mourelo EL (2014). Understanding the Drivers of the Youth Labour Market in Kenya.

[55] Xue L, Zhu B, Wu Y, Wei G, Liao S, Yang C, Wang J, Zhang H, Ren L, Han Q (2019). Dynamic projection of ecological risk in the Manas River basin based on terrain gradients. Sci. Total Environ..

[56] Ives B, Cossick K, Adams D (2019). Amazon Go: Disrupting retail?. J. Inf. Technol. Teach. Cases.

[57] Leavey A, Zwaigenbaum L, Heavner K, Burstyn I (2013). Gestational age at birth and risk of autism spectrum disorders in Alberta, Canada. J. Pediatr..

[58] Yang J, Ren J, Sun D, Xiao X, Xia JC, Jin C, Li X (2021). Understanding land surface temperature impact factors based on local climate zones. Sustain. Cities Soc..

[59] Maryam J, Banday UJ, Mittal A (2018). Trade intensity and revealed comparative advantage: An analysis of Intra-BRICS trade. Int. J. Emerg. Mark..

[60] Yu H, Xie W, Sun L, Wang Y (2021). Identifying the regional disparities of ecosystem services from a supply-demand perspective. Resour. Conserv. Recycl..

[61] Du Y, Deng F, Liao F (2019). A model framework for discovering the spatio-temporal usage patterns of public free-floating bike-sharing system. Transp. Res. Part C Emerg. Technol..

[62] Guo S, Ma H (2021). Does industrial agglomeration promote high-quality development of the Yellow River Basin in China? Empirical test from the moderating effect of environmental regulation. Growth Change.

[63] Yang R, Liu Y, Long H, Qiao L (2015). Spatio-temporal characteristics of rural settlements and land use in the Bohai Rim of China. J. Geogr. Sci..

