# Supplementary material for: Research on the relationship between population distribution pattern and urban industrial facility agglomeration in China
Source: Sci Rep. 2023 Sep 27;13:16225. doi: 10.1038/s41598-023-43376-4 (PMC10533829; doi:10.1038/s41598-023-43376-4)
Supplement: Supplementary file 8 — Supplementary Information 8. [file 41598_2023_43376_MOESM8_ESM.doc]

|  |  |  |  |
| --- | --- | --- | --- |
| 15 Catering Services | 21 Catering Services | 15 Famous tourist sites | 21 Famous tourist sites |
|  |  |  |  |
| 15 Company | 21 Company | 15 Shopping service | 21 Shopping service |
|  |  |  |  |
| 15 Financial insurance service | 21 Financial insurance service | 15 Science, education and cultural services | 21 Science, education and cultural services |
|  |  |  |  |
| 15 Business residence | 21 Business residence | 15 Domestic services | 21 Domestic services |
|  |  |  |  |
| 15 Sports and Leisure Services | 21 Sports and Leisure Services | 15 Healthcare services | 21 Healthcare services |
|  |  |  |  |
| 15 Government agencies and social groups | 21 Government agencies and social groups | 15 Accommodation service | 21 Accommodation service |
| Fig.S1 3D kernel density map of industrial facilities in each city in 2015 and 2021 | | | |

|  |  |  |  |
| --- | --- | --- | --- |
| 15 Catering Services | 21 Catering Services | 15 Famous tourist sites | 21 Famous tourist sites |
|  |  |  |  |
| 15 Company | 21 Company | 15 Shopping service | 21 Shopping service |
|  |  |  |  |
| 15 Financial insurance service | 21 Financial insurance service | 15 Science, education and cultural services | 21 Science, education and cultural services |
|  |  |  |  |
| 15 Business residence | 21 Business residence | 15 Domestic services | 21 Domestic services |
|  |  |  |  |
| 15 Sports and Leisure Services | 21 Sports and Leisure Services | 15 Healthcare services | 21 Healthcare services |
|  |  |  |  |
| 15 Government agencies and social groups | 21 Government agencies and social groups | 15 Accommodation service | 21 Accommodation service |
| Fig.S2 LISA map of industrial facility agglomeration in China in 2015 and 2021 | | | |

|  |  |  |  |
| --- | --- | --- | --- |
| 15 Catering Services | 21 Catering Services | 15 Famous tourist sites | 21 Famous tourist sites |
|  |  |  |  |
| 15 Company | 21 Company | 15 Shopping service | 21 Shopping service |
|  |  |  |  |
| 15 Financial insurance service | 21 Financial insurance service | 15 Science, education and cultural services | 21 Science, education and cultural services |
|  |  |  |  |
| 15 Business residence | 21 Business residence | 15 Domestic services | 21 Domestic services |
|  |  |  |  |
| 15 Sports and Leisure Services | 21 Sports and Leisure Services | 15 Healthcare services | 21 Healthcare services |
|  |  |  |  |
| 15 Government agencies and social groups | 21 Government agencies and social groups | 15 Accommodation service | 21 Accommodation service |
| Fig.S3 Scatter plot of China's urban population growth rate and urban industrial agglomeration in 2015 and 2021 | | | |

| Tab.S1 Notes on "Eastern Region, Central Region and Western Region" | | |
| --- | --- | --- |
| Eastern Region | Central Region | Western Region |
| The eastern region includes Beijing, Tianjin, Hebei, Liaoning, Shanghai, Jiangsu, Zhejiang, Fujian, Taiwan, Shandong, Guangdong, Hainan, Hong Kong Special Administrative Region and Macao Special Administrative Region. | The central region includes Heilong Jiangxi, Jilin, Shanxi, Anhui, Jiangxi, Henan, Hubei and Hunan | The western region includes Inner Mongolia, Guangxi, Chongqing, Sichuan, Guizhou, Yunnan, Tibet, Shaanxi, Gansu, Qinghai, Ningxia and Xinjiang. |

|  |  |  |
| --- | --- | --- |
| 2015 Catering Services | | |
|  |  |  |
| 2018 Catering Services | | |
|  |  |  |
| 2021 Catering Services | | |
|  |  |  |
| 2015 Famous tourist sites | | |
|  |  |  |
| 2018 Famous tourist sites | | |
|  |  |  |
| 2021 Famous tourist sites | | |
|  |  |  |
| 2015 Company | | |
|  |  |  |
| 2018 Company | | |
|  |  |  |
| 2021 Company | | |
|  |  |  |
| 2015 Shopping service | | |
|  |  |  |
| 2018 Shopping service | | |
|  |  |  |
| 2021 Shopping service | | |
|  |  |  |
| 2015 Financial insurance service | | |
|  |  |  |
| 2018 Financial insurance service | | |
|  |  |  |
| 2021 Financial insurance service | | |
|  |  |  |
| 2015 Science, education and cultural services | | |
|  |  |  |
| 2018 Science, education and cultural services | | |
|  |  |  |
| 2021 Science, education and cultural services | | |
|  |  |  |
| 2015 Business residence | | |
|  |  |  |
| 2018 Business residence | | |
|  |  |  |
| 2021 Business residence | | |
|  |  |  |
| 2015 Domestic services | | |
|  |  |  |
| 2018 Domestic services | | |
|  |  |  |
| 2021 Domestic services | | |
|  |  |  |
| 2015 Sports and Leisure Services | | |
|  |  |  |
| 2018 Sports and Leisure Services | | |
|  |  |  |
| 2021 Sports and Leisure Services | | |
|  |  |  |
| 2015 Healthcare services | | |
|  |  |  |
| 2018 Healthcare services | | |
|  |  |  |
| 2021 Healthcare services | | |
|  |  |  |
| 2015 Government agencies and social groups | | |
|  |  |  |
| 2018 Government agencies and social groups | | |
|  |  |  |
| 2021 Government agencies and social groups | | |
|  |  |  |
| 2015 Accommodation service | | |
|  |  |  |
| 2018 Accommodation service | | |
|  |  |  |
| 2021 Accommodation service | | |
| Eastern Region | Central Region | Western Region |
| Fig.S4 The relationship between industrial agglomeration and population growth by dividing China into eastern region, central region, and western region | | |
